# Modified diffusion-weighted imaging-Alberta Stroke Program Early Computed Tomography Score including deep white matter lesions predicts symptomatic intracerebral hemorrhage following intravenous thrombolysis

**DOI:** 10.1007/s11239-019-01979-7

**Published:** 2019-11-19

**Authors:** Koji Tanaka, Shoji Matsumoto, Konosuke Furuta, Takeshi Yamada, Sukehisa Nagano, Kei-ichiro Takase, Taketo Hatano, Ryo Yamasaki, Jun-ichi Kira

**Affiliations:** 1 Department of Neurology, Neurological Institute, Graduate School of Medical Sciences, Kyushu University, 3-1-1 Maidashi, Higashi-ku, Fukuoka, 812-8582 Japan; 2 Department of Neurology, Kokura Memorial Hospital, Kitakyushu, Japan; 3 Department of Comprehensive Strokology, Fujita Health University School of Medicine, Toyoake, Japan; 4 Department of Neurology, Saiseikai Fukuoka General Hospital, Fukuoka, Japan; 5 Department of Neurology, Fukuoka City Hospital, Fukuoka, Japan; 6 Department of Neurology, Iizuka Hospital, Iizuka, Japan; 7 Department of Neurosurgery, Kokura Memorial Hospital, Kitakyushu, Japan

**Keywords:** Symptomatic intracerebral hemorrhage, Thrombolysis, Ischemic stroke, Alberta Stroke Program Early Computed Tomography Score, Diffusion-weighted imaging

## Abstract

The Alberta Stroke Program Early Computed Tomography Score (ASPECTS) is widely used for the assessment of early ischemic changes (EICs) before thrombolysis. However, for symptomatic intracerebral hemorrhage (sICH) following intravenous recombinant tissue plasminogen activator (rt-PA), the prediction abilities of CT-ASPECTS, diffusion-weighted imaging (DWI)-ASPECTS, and DWI-ASPECTS including EICs in deep white matter (DWI-ASPECTS + W) are unclear. We investigated associations between each score and sICH following intravenous rt-PA. Data from consecutive patients who received intravenous rt-PA for acute ischemic stroke from 2005 to 2015 in four hospitals were retrospectively screened. We included data from patients who had undergone both CT and magnetic resonance imaging before thrombolysis and without evidence of posterior circulation stroke. We analyzed the ability of CT-ASPECTS, DWI-ASPECTS, and DWI-ASPECTS + W to predict sICH, accompanied by an increase in the National Institutes of Health Stroke Scale (NIHSS) score of ≥ 4 within the initial 36 h. Of 455 patients (273 men, median 75 years old), sICH occurred in 15 patients (3.3%). Receiver operating characteristics curve analysis showed that the optimal cut-offs of CT-ASPECTS, DWI-ASPECTS, and DWI-ASPECTS + W for predicting sICH were ≤ 9 (sensitivity 60.0%, specificity 59.8%, c-statistic 0.625), ≤ 6 (sensitivity 53.3%, specificity 80.9%, c-statistic 0.718), and ≤ 8 (sensitivity 86.7%, specificity 55.9%, c-statistic 0.756), respectively. A DWI-ASPECTS + W of ≤ 8 was independently associated with sICH (odds ratio 5.21, 95% confidence interval 1.30–35.31) after adjustment for pretreatment with antithrombotic agents, pretreatment NIHSS score, and large artery occlusions. DWI-ASPECTS + W predicted sICH in patients with acute anterior circulation stroke receiving intravenous rt-PA.

## Highlights

- The association between Alberta Stroke Program scores (CT-ASPECTS, DWI-ASPECTS, and DWI-ASPECTS + W) and symptomatic intracerebral hemorrhage (sICH) following intravenous recombinant tissue plasminogen activator (rt-PA) has not been investigated.
- DWI-ASPECTS + W predicted sICH more accurately than CT-ASPECTS and DWI-ASPECTS in patients who received intravenous rt-PA for acute ischemic stroke.
- A DWI-ASPECTS + W of ≤ 8 was independently associated with sICH after adjustment for pretreatment of antithrombotic agents, severity of stroke symptoms, and large artery occlusions.
- Further research should assess the ability of DWI-ASPECTS + W to predict long-term functional outcomes.

## Introduction

Intravenous recombinant tissue plasminogen activator (rt-PA) is an established treatment for acute ischemic stroke that reduces mortality and improves long-term functional outcomes. However, intravenous administration of rt-PA increases the risk of sICH [1, 2], which leads to high mortality and poor functional outcomes [3]. Early ischemic changes (EICs) on pretreatment computed tomography (CT) or diffusion-weighted imaging (DWI) are predictive for the efficacy of intravenous rt-PA [4–6]. The Alberta Stroke Program Early CT Score (ASPECTS) is a standardized scoring system for the assessment of EICs and is widely used in clinical practice for time-dependent treatment of acute ischemic stroke [7, 8]. The scoring method of ASPECTS has also been applied for the measurement of EICs on DWI with good inter-rater agreement [9–13]. A low DWI-ASPECTS has been identified as predictive for sICH following intravenous rt-PA for acute ischemic stroke [10, 11, 14].

Recently, Kawano et al. [15] established a modified scoring method, the 11-point DWI-ASPECTS + W, which includes a score of 1 for EICs in deep white matter (WM) in addition to each of the 10 original ASPECTS regions (caudate nucleus, lentiform nucleus, internal capsule, insular ribbon, and M1–6 cortical regions of the middle cerebral artery [MCA]). In that study, both a low DWI-ASPECTS + W and administration of intravenous rt-PA were predictive for any ICH in patients with acute ischemic stroke, while CT-ASPECTS and DWI-ASPECTS were not. However, in that study, only 36 of 164 patients (22%) received intravenous rt-PA, and no patients developed sICH following thrombolysis. Therefore, evidence for the association between DWI-ASPECTS + W and sICH following intravenous rt-PA has been inconclusive. Thus, the purpose of this study was to investigate the ability of CT-ASPECTS, DWI-ASPECTS, and DWI-ASPECTS + W to predict sICH following intravenous rt-PA. We hypothesized that DWI-ASPECTS + W would more accurately predict sICH in patients receiving intravenous rt-PA than CT-ASPECTS and DWI-ASPECTS.

## Methods

### Subjects

We used data from multicenter retrospective observational study that has been described in detail elsewhere [16]. Briefly, the present study was conducted using data from four urban emergency hospitals with a stroke unit (Saiseikai Fukuoka General Hospital, Fukuoka City Hospital, Iizuka Hospital, and Kokura Memorial Hospital). Subjects in this study comprised consecutive patients who received intravenous rt-PA for acute ischemic stroke between 1 October 2005 and 31 December 2015. All patients received intravenous administration of 0.6 mg/kg alteplase in accordance with the Japanese guidelines [17]. This study was approved by the Ethics Committees of Kyushu University Hospital (29-111) and each of the facilities.

This study enrolled patients who underwent both CT and magnetic resonance imaging (MRI) including DWI prior to the administration of rt-PA and without evidence of posterior circulation stroke, including EICs in the territory of the vertebrobasilar arteries or posterior cerebral artery and/or occlusion of the basilar artery.

The following clinical information was systematically extracted from medical records: age, sex, vascular risk factors (hypertension, diabetes mellitus, and dyslipidemia), atrial fibrillation, history of stroke, and pretreatment of antithrombotic agents. The pretreatment systolic and diastolic blood pressure, severity of stroke symptoms as assessed by the National Institutes of Health Stroke Scale (NIHSS) score, and onset-to-treatment time were obtained from emergency medical charts. sICH was defined as CT evidence of new parenchymal ICH associated with neurological deterioration that corresponded to an increase of ≥ 4 points from the baseline NIHSS score within 36 h after the treatment.

### Assessment of ASPECTS

Before the intravenous administration of rt-PA, MRI was performed, usually after CT. The time of starting CT and MRI was collected from the electronic time stamp of when the first sequence was acquired. CT scans were performed according to a standard multi-slice CT scan protocol (without contrast enhancement, 120 kV, 200–270 mA, 0.75- to 2-s scan time, 5-mm slice thickness, inferior orbitomeatal baseline, and obtained at an appropriate window width and level setting of ≥ 95 Hounsfield units). MRI scans were performed on a 1.5 T scanner. MRI protocols were not entirely uniform between hospitals, but all included axial DWI that used single-shot echoplanar imaging (b value, 1000 s/mm^2^; slice thickness, 5–7.5 mm; inter-slice gap, 1–1.5 mm).

CT-ASPECTS and DWI-ASPECTS were calculated according to the 10-item ASPECTS lesion method. The 11-point DWI-ASPECTS + W was also calculated by assigning a score of 1 for normal and a score of 0 for EICs in the WM (defined as hyperintense lesions in the corona radiata) in addition to the 10 original ASPECTS regions. The outermost limit of the WM lesion was the subcortex of the M5 region and the innermost limit was the caudate nucleus, which were evaluated at the level 2 cm superior to the thalamus and striatum, as previously described [15, 18]. For each score, two experienced physicians (K Tanaka and one of the following: SM, TY, SN, or K Takase) independently and retrospectively calculated the CT-ASPECTS, DWI-ASPECTS, and DWI-ASPECTS + W for each hospital. During the imaging interpretations, physicians were blinded to the patients’ clinical information and the outcome of the imaging findings. Disagreements were resolved by consensus.

Arterial occlusion sites were assessed using MR angiography, carotid ultrasonography, and/or CT angiography. Large artery occlusions were defined as occlusions of the internal carotid artery or proximal portion of the MCA detected by any modality.

### Statistical analysis

All statistical analyses were performed using JMP statistical software version 9.0 (SAS Institute, Inc., Cary, NC, USA). Data are expressed as the median and interquartile range for continuous variables and the count and percentage for categorical variables. The kappa statistics were used to assess the investigators’ agreement about CT-ASPECTS, DWI-ASPECTS, and WM lesions. Relationships between CT-ASPECTS, DWI-ASPECTS, and DWI-ASPECTS + W were assessed using a Bland–Altman plot. To obtain the optimal CT-ASPECTS, DWI-ASPECTS, and DWI-ASPECTS + W cut-offs required to predict sICH, receiver operating characteristics curves were constructed. The c-statistics (area under the receiver operating characteristics curve) for the three scores were compared using a nonparametric method [19]. Clinical characteristics between patients with and without sICH were compared using the Chi squared test, Fisher’s exact test, or Wilcoxon rank sum test as appropriate. Multivariate logistic regression analysis was performed to assess the independent impact of each cut-off on sICH. Variables with a *p* value of < 0.05 in the univariate analysis were included in the multivariate model. A p-value of < 0.05 was considered statistically significant.

## Results

Overall, 750 consecutive patients who received intravenous rt-PA during the study period were included; of these, 295 were excluded because they had received intravenous rt-PA twice during the study period (n = 2), did not undergo a CT or MRI study (n = 225), had missing data (n = 1), or exhibited evidence of posterior circulation stroke (n = 67), including EICs in the thalamus (n = 12), cerebellum (n = 10), brain stem (n = 18), posterior cerebral artery territory (n = 8), and/or occlusion of the basilar artery (n = 33). Finally, data from 455 patients (273 men, median 75 years old) were included in the analysis. The median time from onset of stroke to CT, time delay of MRI after CT, and onset to treatment time was 65 (49–98) min, 23 (14–33) min, and 141 (113–173) min, respectively.

Antithrombotic agents were prescribed to 171 patients (37.6%) prior to stroke. Antiplatelet therapy (aspirin [n = 81], clopidogrel [n = 12], cilostazol [n = 12], and dual antiplatelet therapy [n = 12]) was prescribed to 117 patients. Anticoagulant therapy (warfarin [n = 71], direct oral anticoagulants [n = 3], and unfractionated heparin [n = 3]) was prescribed to 77 patients. Twenty-three patients received a combination of antiplatelet and anticoagulant therapy. The median CT-ASPECTS, DWI-ASPECTS, and DWI-ASPECTS + W scores were 10 (9–10), 8 (7–9), and 9 (8–10), respectively. WM lesions were seen in 255 patients (56.0%), and the CT-ASPECTS and DWI-ASPECTS scores were lower in patients with WM lesions (10 [8–10] vs. 10 [9–10], p = 0.003 and 8 [7–10] vs. 9 [8–10], p < 0.001, respectively). Large artery occlusions were seen in 227 (51.0%) of 445 patients who were evaluated for arterial occlusion sites before thrombolysis, including the internal carotid artery (n = 86) and the proximal portion of the MCA (n = 141). Sixty-three patients (13.8%) underwent endovascular therapy alongside thrombolysis.

**Fig. 1:**
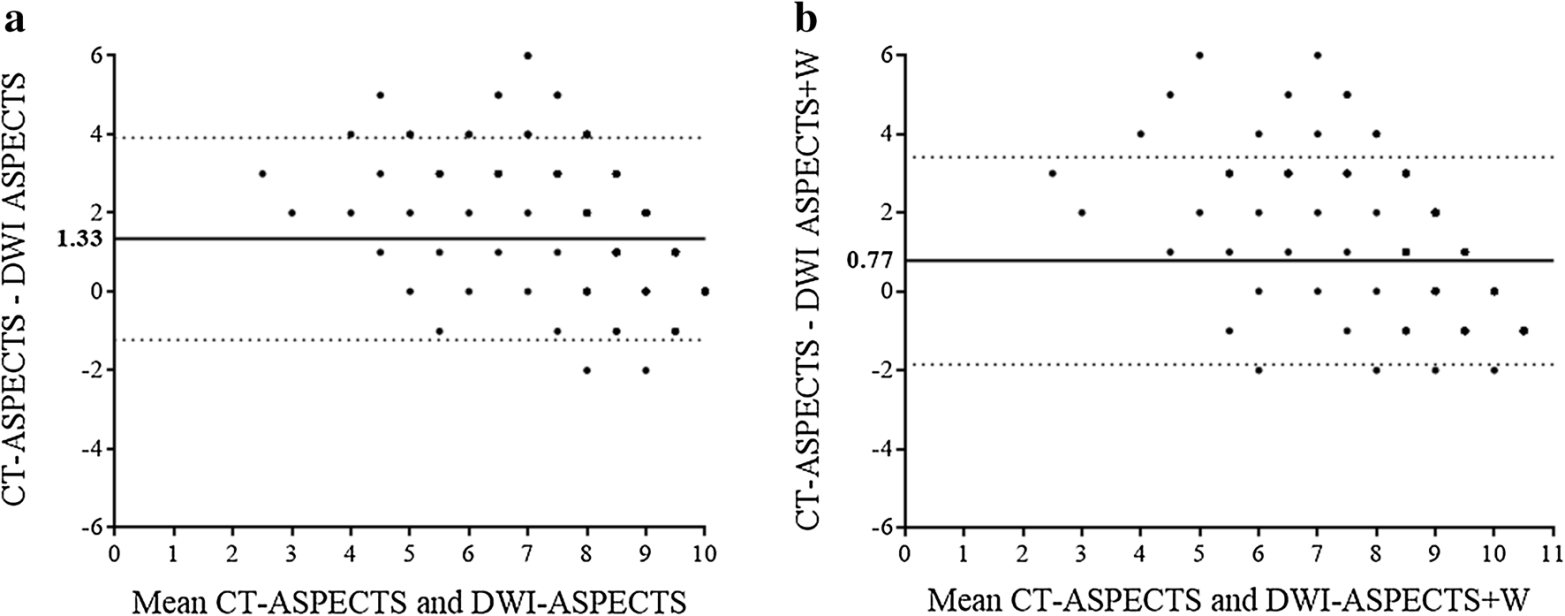
Bland–Altman plots of Alberta Stroke Program scores. **a** The mean difference between CT-ASPECTS and DWI-ASPECTS was 1.33. **b** The mean difference between CT-ASPECTS and DWI-ASPECTS + W was 0.77. The horizontal line shows the mean difference in scores, and the dotted lines show the standard deviations. *ASPECTS* Alberta Stroke Program Early Computed Tomography Score, *DWI* diffusion-weighted imaging

The weighted kappa statistics of agreement between the physicians for CT-ASPECTS and DWI-ASPECTS were 0.482 and 0.696, respectively. The kappa statistic for the presence of WM lesions was 0.618. Figure 1 shows the Bland–Altman plots of differences between CT-ASPECTS vs. DWI-ASPECTS and CT-ASPECTS vs. DWI-ASPECTS + W. The mean difference between CT-ASPECTS and DWI-ASPECTS was 1.33, and the mean difference between CT-ASPECTS and DWI-ASPECTS + W was 0.77.

**Fig. 2:**
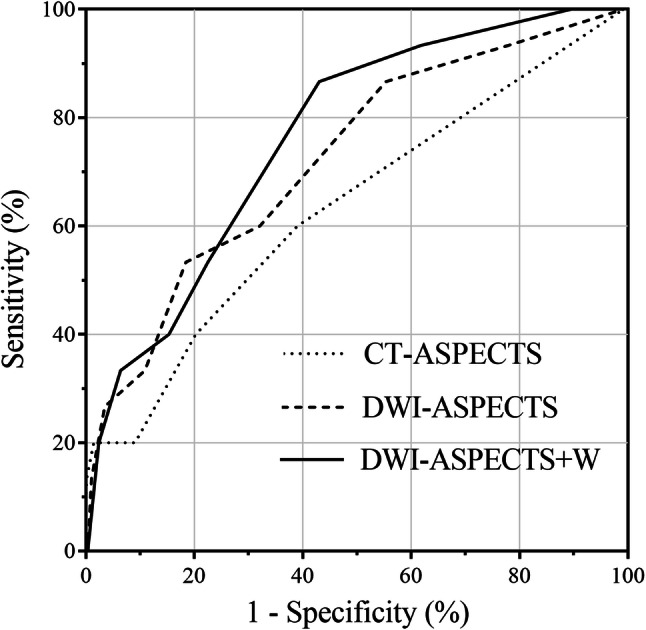
Receiver operating characteristics curves of Alberta Stroke Program scores for predicting symptomatic intracerebral hemorrhage. There was a significant difference in the c-statistics among the three scores (p = 0.039), and DWI-ASPECTS + W was superior to CT-ASPECTS (p = 0.032) and DWI-ASPECTS (p = 0.040) in predicting symptomatic intracerebral hemorrhage. *ASPECTS* Alberta Stroke Program Early Computed Tomography Score, *DWI* diffusion-weighted imaging

Of the 455 patients, 15 (3.3%) had sICH. Compared with patients without sICH, patients with sICH more frequently received pretreatment with antithrombotic agents (66.7% vs. 36.6%, p = 0.027), had a higher pretreatment NIHSS score (median 21 vs. 15, p = 0.003), and more frequently had large artery occlusion (80.0% vs. 50.0%, p = 0.033). The DWI-ASPECTS (median 6 vs. 8, p = 0.004) and DWI-ASPECTS + W (median 7 vs. 9, p < 0.001) scores were lower in patients with sICH than those without (Table 1). For predicting sICH, the optimal cut-offs of CT-ASPECTS, DWI-ASPECTS, and DWI-ASPECTS + W were ≤ 9 (sensitivity 60.0%, specificity 59.8%, c-statistic 0.625), ≤ 6 (sensitivity 53.3%, specificity 80.9%, c-statistic 0.718), and ≤ 8 (sensitivity 86.7%, specificity 55.9%, c-statistic 0.756), respectively (Table 2). There was a significant difference in the c-statistic among the three scores (p = 0.039), and DWI-ASPECTS + W was superior to CT-ASPECTS (p = 0.032) and DWI-ASPECTS (p = 0.040) for predicting sICH (Fig. 2).

**Table 1:** Univariate analysis of clinical characteristics of patients with and without symptomatic intracerebral hemorrhage

Variable	Total N = 455	With sICH N = 15	Without sICH N = 440	p-value
Sex, male*	273 (60.0)	9 (60.0)	264 (60.0)	1.000
Age (years)†	75 (65–82)	76 (67–87)	75 (65–82)	0.481
Hypertension*	308 (67.7)	10 (66.7)	298 (67.7)	1.000
Dyslipidemia*	121 (26.6)	4 (26.7)	117 (26.6)	1.000
Diabetes mellitus*	98 (21.5)	6 (40.0)	92 (20.9)	0.104
Atrial fibrillation*	243 (53.4)	10 (66.7)	233 (53.0)	0.431
Pretreatment of antithrombotic agents*	171 (37.6)	10 (66.7)	161 (36.6)	0.027
Systolic blood pressure (mmHg)†	159 (141–180)	158 (150–171)	159 (140–180)	0.738
Diastolic blood pressure (mmHg)†	86 (74–100)	84 (76–94)	86 (74–100)	0.854
Pretreatment NIHSS score†	16 (10–21)	21 (17–26)	15 (10–20.75)	0.003
CT-ASPECTS†	10 (9–10)	10 (8–10)	10 (9–10)	0.064
DWI-ASPECTS†	8 (7–9)	6 (4–8)	8 (7–9)	0.004
DWI-ASPECTS + W†	9 (8–10)	7 (5–8)	9 (8–10)	< 0.001
Large artery occlusions (N = 445)*	227 (51.0)	12 (80.0)	215 (50.0)	0.033
Onset to treatment time (min)†	141 (113–173)	130 (100–164)	141 (113–174)	0.507
Endovascular therapy*	63 (13.8)	0 (0)	63 (14.3)	0.243

Data are presented as N (%) or median (interquartile range)

*NIHSS* National Institutes of Health Stroke Scale, *ASPECTS* Alberta Stroke Program Early Computed Tomography Score, *DWI* diffusion weighted imaging

*Fisher’s exact test, ^†^Wilcoxon rank sum test

**Table 2:** Receiver-operating-characteristic curve analysis of Alberta Stroke Program scores for predicting symptomatic intracerebral hemorrhage

Variable	c-statistic (95% CI)	Optimal cutoff	Sensitivity (%)	Specificity (%)	PPV (%)	NPV (%)
CT-ASPECTS	0.625 (0.468–0.759)	≤ 9	60.0	59.8	3.6	97.8
DWI-ASPECTS	0.718 (0.565–0.833)	≤ 6	53.3	80.9	8.7	98.1
DWI-ASPECTS + W	0.756 (0.629–0.850)	≤ 8	86.7	55.9	6.3	99.2

*ASPECTS* Alberta Stroke Program Early Computed Tomography Score, *CI* confidence interval, *PPV* positive predictive value, *NPV* negative predictive value, *DWI* diffusion weighted imaging

The multivariate logistic regression analysis revealed that a DWI-ASPECTS + W of ≤ 8 was significantly associated with sICH (odds ratio 5.21, 95% confidence interval 1.30–35.31) after adjustment for pretreatment of antithrombotic agents, pretreatment NIHSS score, and large artery occlusions (Table 3).

**Table 3:** Multivariate logistic regression analysis of cut-offs of Alberta Stroke Program scores for predicting symptomatic intracerebral hemorrhage

Variable	Odds ratio (95% CI)	p-value
CT-ASPECTS ≤ 9	1.25 (0.39–3.81)	0.694
DWI-ASPECTS ≤ 6	2.72 (0.89–8.56)	0.079
DWI-ASPECTS + W ≤ 8	5.21 (1.30–35.31)	0.018

*ASPECTS* Alberta Stroke Program Early Computed Tomography Score, *CI* confidence interval, *DWI* diffusion weighted imaging

The models were adjusted for pretreatment of antithrombotic agents, pretreatment National Institutes of Health Stroke Scale score, and large artery occlusions

## Discussion

This study investigated the association between the extent of EIC as measured by three scores and sICH following intravenous rt-PA. Among the three scores, DWI-ASPECTS + W was superior to both CT-ASPECTS and DWI-ASPECTS in predicting sICH. This study showed that the inter-rater agreement was good for DWI-ASPECTS and moderate for CT-ASPECTS. This better inter-rater agreement on DWI than CT is consistent with that in previous studies [9, 11, 12, 13]. The mean DWI-ASPECTS was 1.33 points lower than the mean CT-ASPECTS. This discrepancy between DWI-ASPECTS and CT-ASPECTS scores was larger than in previous studies, which reported a mean difference of 0.43 [20] in ischemic stroke patients within 7 h of onset, a median difference of 1 point [21] in stroke patients within 24 h of onset, and a mean difference of 0.92 in stroke patients within 3 h of onset [11]. There may be two possible reasons for this finding. First, the median time from stroke onset to CT in this study was 65 min, which was relatively shorter than that in the previous studies; the mean and median times from symptom onset to CT were 117 min and 2.7 h [20, 21]. Therefore, some EICs might have been overlooked before becoming apparent on CT. Second, the median time delay of DWI after CT was 23 min, which was slightly longer than that of 19 min previously reported in a study with patients who had received intravenous rt-PA [11]. Alongside the higher sensitivity of DWI than CT for detecting EICs, some EICs became apparent after CT.

This study showed that the c-statistics for DWI-ASPECTS + W are higher than for CT-ASPECTS and DWI-ASPECTS for predicting sICH following intravenous rt-PA. This indicates that WM lesions, which are often difficult to detect on CT, can be a precursor to the development of sICH. The corona radiata consists of various WM tracts and is mainly fed by the deep lenticulostriate branches of the MCA [22]. Subcortical WM has higher thresholds for infarctions than does gray matter [23–25]. Therefore, WM lesions indicate more severe ischemia in the MCA territory. This is also supported by our finding that patients with WM lesions had lower CT-ASPECTS and DWI-ASPECTS than those without WM lesions. Kawano et al. [18] reported that the presence of WM lesions was predictive for the absence of early dramatic improvement after intravenous rt-PA. An increased risk of sICH in patients with WM lesions would partially explain the negative correlation between WM lesions and early dramatic improvement.

The cut-off value of DWI-ASPECTS + W was independently associated with sICH following intravenous rt-PA. The cut-off value, a DWI-ASPECTS + W of ≤ 8, was 1 point higher than that reported in a previous study [15], in which the DWI-ASPECTS + W cut-off value for predicting any ICH was ≤ 7. This might be because all participants in this study received intravenous rt-PA, and the elevated risk of bleeding lowered the threshold of sICH. Our results indicate that patients with a DWI-ASPECTS + W of ≥ 9 can be administered rt-PA safely. Although recent American Heart Association guidelines recommend non-contrast CT as an imaging modality prior to intravenous rt-PA [26], and the utility of CT cannot be denied, our results indicate that DWI is more useful for predicting sICH following intravenous rt-PA. Moreover, the 11-point DWI-ASPECTS + W, which simply adds information on WM lesions to the 10 ASPECTS regions, was able to more accurately predict which patients were prone to sICH in the acute phase than the original ASPECTS based on both CT and DWI.

This study has several limitations. First, this was a retrospective study with a limited number of patients, which might have led to statistical errors. Second, the imaging modality before thrombolysis was decided by an independent attending physician. This might contribute to selection bias in patients who underwent both CT and DWI. Moreover, performing both CT and DWI before thrombolysis might lead to some delay in treatment and affect the outcome. Third, post-treatment parameters that could have affected the incidence of sICH (e.g. blood pressure, recanalization of the occluded artery, and antithrombotic agents used after the initial 24 h) were not included. Further research including these factors will inform the development of preventative measures for sICH following intravenous rt-PA in patients with low ASPECTS. Finally, associations between each ASPECTS and long-term functional outcomes were unclear because we did not use the 3-month modified Rankin scale in the present study.

In conclusion, DWI-ASPECTS + W was able to predict sICH following intravenous rt-PA more accurately than CT-ASPECTS and DWI-ASPECTS. DWI-ASPECTS + W is a promising method for predicting sICH following intravenous rt-PA in patients with anterior circulation stroke.
